# Continence Across Continents To Upend Stigma and Dependency (CACTUS-D): study protocol for a cluster randomized controlled trial

**DOI:** 10.1186/s13063-015-1099-x

**Published:** 2015-12-10

**Authors:** Cara Tannenbaum, Eleanor van den Heuvel, Xavier Fritel, Kenneth Southall, Jeffrey Jutai, Saima Rajabali, Adrian Wagg

**Affiliations:** Institut Universitaire de Gériatrie de Montréal, Faculté de Médecine, Université de Montréal, Montréal, QC Canada; Brunel Institute for Ageing Studies, Brunel University, Uxbridge, UK; Faculty of Medicine and Pharmacy, University of Poitiers, Poitiers, France; École d’Orthophonie et d’Audiologie, Université de Montréal, Montréal, QC Canada; Interdisciplinary School of Health Sciences, University of Ottawa, Ottawa, ON Canada; Division of Geriatric Medicine, University of Alberta, Edmonton, AB Canada

**Keywords:** Urinary continence, Stigma, Falls, Cost, Community-dwelling older women, Quality of life, Randomized controlled trial, Public health

## Abstract

**Background:**

Urinary incontinence occurs in 40 % of women aged 65 years and over; however, only 15 % seek care and many delay healthcare seeking for years. Incontinence is associated with depression, social isolation, reduced quality of life, falls and other comorbidities. It is accompanied by an enormous cost to the individual and society. Despite the substantial implications of urinary incontinence on social, psychological and physical well-being of older women, the impact of continence promotion on urinary symptom improvement and subsequent effects on falls, quality of life, stigma, social participation and the cost of care remains unknown.

**Methods:**

This study is a mixed methods multi-national open-label 2-arm parallel cluster randomized controlled trial aiming to recruit 1000 community-dwelling incontinent women aged 65 years and older across Quebec, Western Canada, France and United Kingdom. Participants will be recruited through community organizations. Data will be collected at 6 time points: baseline and 1 week, 3 months, 6 months, 9 months and 12 months after baseline. One of the primary objectives is to evaluate whether the continence promotion intervention improves incontinence symptoms (measured with the Patient Global Impression of Improvement questionnaire, PGI-I) at 12 months post intervention compared to the control group. Other co-primary outcomes include changes in incontinence-related stigma, fall reduction, and incremental cost-effectiveness ratio and quality-adjusted life years. Data analysis will account for correlation of outcomes (clustering) within community organizations. A qualitative sub-study will explore stigma reduction.

**Discussion:**

Community-based continence promotion programs may be a cost-effective strategy to reduce urinary incontinence, stigma and falls among older women with untreated incontinence, and simultaneously improve quality of life and healthy active life expectancy.

**Trial registration:**

ClinicalTrials.gov: NCT01858493, registered 13 May 2013

## Background

The “taboo” syndrome of urinary incontinence has traditionally been stigmatized among older women despite its remarkably high prevalence. In Canada, the United Kingdom (UK) and France, almost 40 % of women aged 65 years and older experience urinary incontinence, but little more than 15 % seek care [1–4]. For many of these women, incontinence symbolizes a decline into frailty and dependency [5]. Coping strategies include the avoidance of social activities because of the potential for embarrassment due to loss of bladder control in public, resulting in a vicious cycle of reduced participation, social isolation, depression and loss of independent living [6]. Urinary incontinence has consistently been associated with shame [6], poor quality of life [6, 7], poor self-rated health [7], social isolation [6], and depression [6, 8].

Incontinence is also linked to falls in older people, especially when leakage is associated with urgency and rushing to the toilet in order to prevent leakage [9–14]. Urge incontinence describes incontinence associated with feelings of urgency, and has been shown to increase the risk of falls by 50 % [9], with resultant fall-related injuries. The relationship between falls and urinary incontinence can be explained by shared underlying pathophysiological mechanisms, problems with dual-tasking and cortical inhibition, and white matter hyperintensities [15–17]. Urinary incontinence alone, and because of its association with falls, has been associated with functional decline, nursing home admission and significant out-of-pocket expenses [18, 19]. This is a tragedy as incontinence is largely treatable, with cures and improvements obtainable at all ages even with conservative management [20].

Receipt of care for incontinence has been shown to lead to measurable improvements in quality of life for older adults [21]. Unfortunately, almost 85 % of incontinent older women never talk to a healthcare practitioner or seek help for their condition [4, 22–24]. Women who do not seek help may consider their incontinence as not being serious enough, have inappropriate beliefs related to incontinence being a normal part of aging, or may imagine that there is nothing that can be done to treat it [24, 25].

To date, few public health initiatives have targeted continence promotion, despite its substantial social, psychological and physical ramifications for older adults. Continence promotion involves challenging inappropriate beliefs about incontinence, educating people that evidence-based therapeutic options exist, and promoting improvements and cures at all ages.

A pilot study compared the effectiveness of three experimental continence promotion interventions against a control intervention on urinary symptom improvement in 259 women aged 60 years and older with untreated incontinence [26]. Participants were recruited from community organizations [27]. The highest rate of urinary symptom improvement occurred among participants who received a combined a 1-hour constructivist learning workshop and a self-management program. Sixty-six percent of participants in this group achieved improvement in urinary symptoms, compared to 11 % of the control group (prevalence difference 55 %, 95 % confidence interval (CI) 43 –67 %, intracluster correlation 0). The number-needed-to-treat was 2 to achieve any improvement in incontinence symptoms, and 5 to attain significant improvement. Changes in knowledge and self-reported risk-reduction behaviors paralleled rates of improvement [26].

The trial described in this protocol seeks to build on the successful pilot study by testing whether the combined continence promotion intervention can diminish incontinence-related stigma and falls, and improve quality of life in addition to reducing urinary symptoms in older community-dwelling women with incontinence. This cluster randomized controlled trial, conducted internationally across the UK, France and Canada, will enable accrual of a larger sample of culturally diverse women in whom to test the continence promotion intervention. Prolonging the follow-up period to 1 year, as compared to only 3 months in the pilot project, will allow us to see if the benefits of the combined intervention can be maintained and improved over 1 year. Further, this will permit time for women to seek healthcare after they have tried some self-management tools. The impact on fall reduction, quality of life and healthy active life expectancy will also be assessed.

### Study hypothesis

**Fig. 1:**
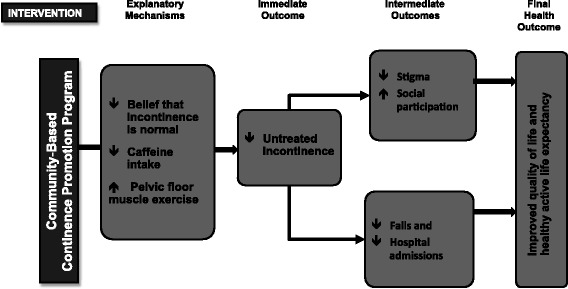
Logic model for the Continence Across Continents To Upend Stigma and Dependency (CACTUS-D) trial

We hypothesize that, compared to the control group, receipt of the combined continence promotion intervention will result in a significant improvement in incontinence symptoms in at least 20 % of women, a reduction in falls of 30 %, an expected change of 4.74 points on the Incontinence Quality of Life Scale (I-QOL) and a gain in utility points on the 6 dimensional derivative of the 12-item Short Form Health Survey (SF-6D) of 0.0126, with which to calculate cost-effectiveness, cost-utility and gains in healthy active life expectancy (Fig. 1).

### Aim and study objectives

This study aims to evaluate the effectiveness of a continence promotion program on incontinence symptoms, rate of falls, self-stigma and quality of life in community-dwelling women with incontinence. In addition, the study aims to evaluate the economic efficiency (incremental cost-effectiveness and cost-utility) of providing community-based group continence workshops to untreated older women with incontinence.

The objectives are to:

1. Evaluate whether the continence promotion intervention yields significant improvement in incontinence symptoms at 1 year post intervention compared to the control group.
2. Examine the effects of a continence promotion intervention on self-stigma among women with untreated urinary incontinence.
3. Examine whether the receipt of a continence promotion intervention leads to a reduction in falls and fall-related injuries.
4. Investigate the effects on quality of life and ascertain the cost-effectiveness/cost-utility of the continence promotion program for senior community-dwelling women.

## Methods/Design

### Design

The Continence Across Continents To Upend Stigma and Dependency (CACTUS-D) study is an open-label, two-arm, parallel group, multi-national cluster randomized controlled trial to be conducted across four sites. A cluster design was deemed necessary to minimize the chances of contamination between participants in different arms of the trial. Individuals in a given community often share information through discussion or direct observation. We surmised that older women rarely migrate to more than one community, hence we were confident that community-level cluster randomization would minimize the chances of contamination between the two arms of the trial.

**Fig. 2:**
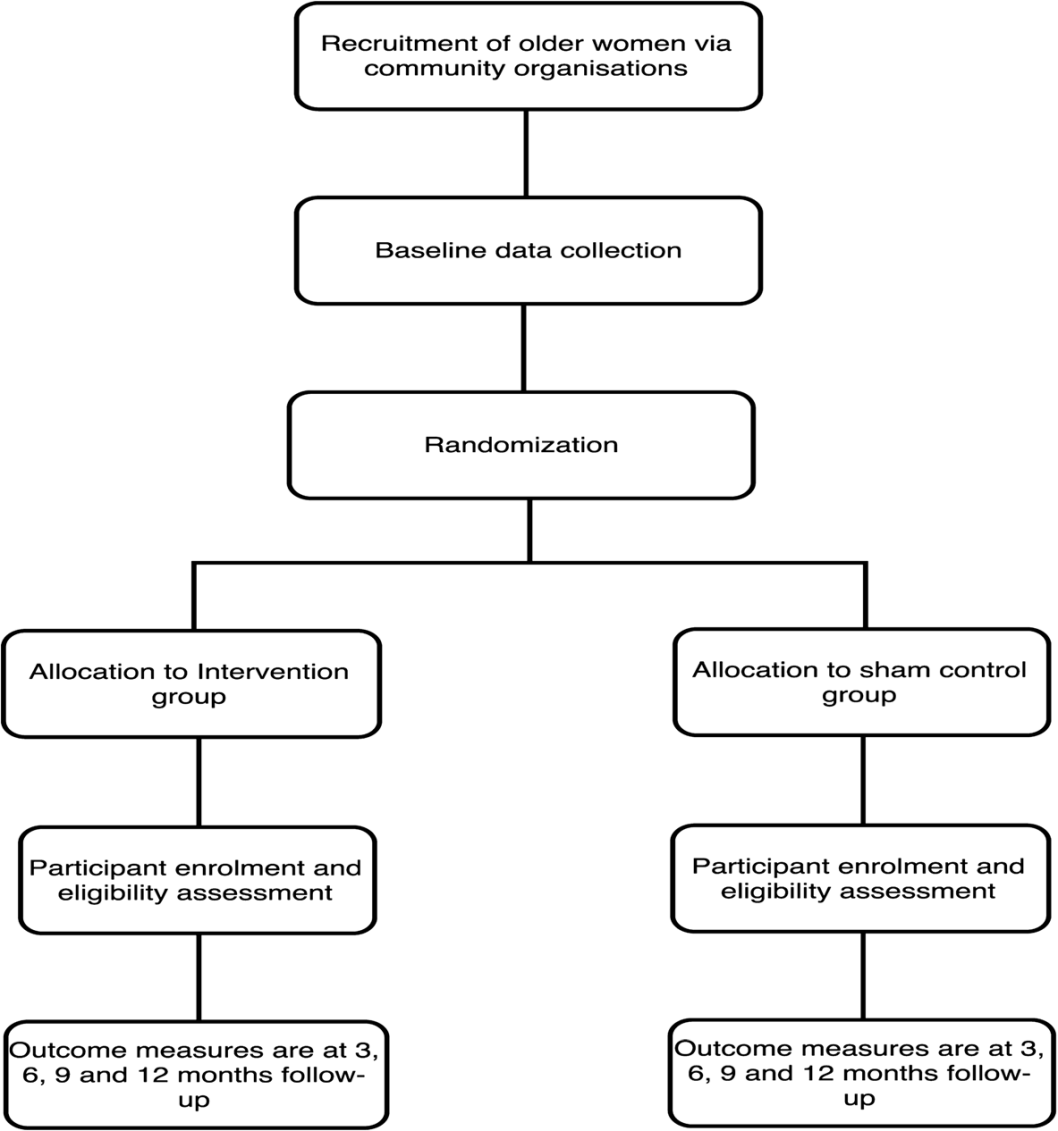
Study flow

The two arms in this trial are the continence promotion intervention arm and the sham control arm. Figure 2 shows the flow of participants through the study. A qualitative study relating to stigmatization associated with continence will be embedded in the trial with a sub-group of participants. The qualitative sub-study will be conducted across all four sites and will seek to understand the effects of a continence promotion intervention on self-stigma among women with untreated urinary incontinence.

An economic evaluation will be conducted alongside the trial to examine the difference in costs and outcomes between the continence promotion intervention and control groups. If the continence promotion intervention is deemed effective in both cost saving and improvement in outcomes, then it is labeled dominant to the comparator. An economic evaluation is useful for decision-making in comparing the cost and outcomes.

### Study participants and setting

#### Eligibility of community organizations

All community organizations in Quebec, Western Canada, France and the UK will be eligible to participate and recruit older community-dwelling women to the trial.

#### Eligibility of community-dwelling participants

Community-dwelling incontinent older women attending workshops organized by participating community organizations will be eligible to take part in the study. Women eligible for inclusion will be those aged 65 years or older who are able to communicate either in English or in French. They will have had to experience urinary incontinence with a frequency of two or more times per week, and not sought professional advice for incontinence symptoms within the past year. Exclusion criteria are women diagnosed with dementia.

### Ethical approval

The ethics review boards of the Institut Universitaire de Gériatrie de Montréal in Quebec, the University of Brunel in the UK, the University of Poitiers in France, and the University of Alberta in Alberta all approved the study protocol by 26 March 2013 (ClinicalTrials.gov identifier: NCT01858493). No personal identifiers will be used in any reports or publications. Study procedures and benefits will be explained to the enrolled women, and written consent will be obtained for baseline measurements.

### Recruitment and application of eligibility criteria

#### Organizations

Recruitment will be via community organizations. Eligible organizations will be contacted via post, telephone or Email by a research assistant, and invited to host an older women’s health workshop. Organizations will be strategically selected by convenience sampling and word of mouth to target older women. Previous organizations from the pilot study [26, 27] for this project will not be recruited. In order to be inclusive of diversity, a conscious effort will be made to approach culturally and socio-economically diverse groups of women through outreach to different community organizations in urban and rural areas from a wide area around each study site. Once the organization has consented to host a workshop and a local organizer has been selected from the organization, a mutually convenient date will be chosen for the local event. The event will be advertised via telephone calls, word of mouth, and targeted advertisements featured in online or paper newsletters, and mailed invitations, printed by the organization inviting their members to attend.

#### Participants

Following the recruitment of community organizations, all women who decide to attend the workshops will be self-identified for participation in the study. Recruitment strategies vary slightly per site, based on the requirements of the respective ethics committees. In France and in Western Canada, baseline data will be collected and participants will be screened and enrolled to the study prior to the receipt of the intervention. In the UK and Quebec, women will complete the baseline questionnaire prior to the delivery of the intervention, but interested participants will sign the consent form and return the baseline questionnaire only after the workshop is completed. In a second screening stage, a research assistant confirms eligibility status and interest in enrolling in the trial via telephone follow-up within 1 week of the workshop.

Across all sites, a subsample of participants will be recruited for an ethnographic study that aims to explore continence stigma, aging, and participant perspectives of the intervention and control workshops. Data from the qualitative interviews will help the team understand the mechanisms and outcomes of the intervention on self-stigma related to incontinence. Approximately 20 participants will be interviewed within 2 weeks of the workshop to explore participant perspectives of the intervention and control workshops, and related feelings associated with continence and age-related stigma. A second group of 20 participants will be interviewed 1 year after the intervention workshop. Five participants from each trial site will be purposively selected from those who have shown significant benefit from the continence promotion intervention, to investigate the impact on stigma and to understand the mechanisms behind the perceived benefits of the workshop.

### Randomization

#### Randomization/Concealment of allocation

Randomization will occur prior to delivery of the intervention. The unit of randomization will be the community organization. Using block randomization with an alternating block size of 2 or 4, we will randomly allocate clusters (via a 1:1 ratio and stratified by site) to the intervention or control group via a computer-generated random digit performed by an independent statistician (Fig. 2). Stratification by site will aim to account for any variation resulting from site characteristics and participants from individual community organizations. The trial is, nonetheless, considered open-label because both the research assistant who delivers the interventions and the study participants who will have received the interventions will be aware of group allocation at the time of implementation.

#### Blinding

The trial is blinded at the level of cluster randomization: community organizations are aware that their participants will receive one of the two interventions, but they will be blinded to group allocation. Participants will be aware of which intervention they receive as the intervention is open-label. A research assistant, who will be blinded to group allocation, will inform, deliver and complete the questionnaires after each telephone follow-up. A statistician blinded to the allocation will conduct the analysis and reporting of the co-primary outcome measures.

### Intervention

The continence promotion intervention is a single 45-minute interactive group continence promotion program delivered by a trained research assistant/workshop facilitator. The continence intervention incorporates constructivist learning and discusses the risk factors, causes and treatments for incontinence and challenges the misconception that incontinence is a normal part of aging. It aims to motivate community-dwelling women to initiate evidence-based self-management and/or to consult for treatment [26–30]. The intervention workshop is introduced as an educational workshop addressing “taboo” topics related to aging (memory problems and incontinence). Risk factors and different etiologies for incontinence are reviewed, physiology and pathology explained in lay terms and modifiable strategies for risk reduction are recommended. A paper-based self-management tool is provided at the end of the workshop to help participants monitor their symptoms before and after attempting evidence-based self-management strategies. This paper-based tool asks women to identify one of the risk factors they may have for urine leakage and to implement a strategy for risk reduction. This is the same intervention that was tested in the pilot trial [26].

The sham control intervention (the comparator) for this study is a single 45-minute interactive group workshop on other health topics of importance to older women. The topics discussed are memory problems, medication interactions, sleep problems, and hearing loss. Incontinence is mentioned briefly during the sham control intervention in order to integrate it with the questionnaire that revolves around bladder problems.

A fidelity protocol and checklist for delivery of the intervention will be applied every 6 months to each study site, in order to ensure standardized implementation of the intervention in each country.

### Study follow-up

Study follow-up will include five telephone calls. The first within a week of the workshop then at, 3, 6, 9 and 12 months post workshop. Telephone interviews normally last from 5 to 40 minutes.

### Outcomes

#### Improvement in urinary incontinence at 12 months

The co-primary outcome will be the participant’s global impression of improvement in incontinence symptoms, measured at 1-year post intervention by telephone interview using the Patient’s Global Impression of Improvement (PGI-I) questionnaire. The PGI-I is a validated, single-item global rating of change scale that asks the patient to describe how their incontinence condition is now compared to how it was prior to the intervention (very much better, much better, a little better, no change, a little bit better, much worse and very much worse) [31]. Significant improvement will be defined as participants rating their incontinence symptoms as much better or very much better. This measure showed excellent acceptability, known-group validity, reliability and responsiveness during the pilot study [26]. The PGI-I questionnaire will be administered at 3 months, 6 months and 1 year post intervention to all participants in all countries by the research assistant at each site (Table 1).

**Table 1:** Timeline of study assessment and data collection

Questionnaires	Baseline	1 week	3 months	6 months	9 months	12 months
	T0	T1	T2	T3	T4	T5
	Pre-workshop	Telephone follow-up and enrollment confirmation	Telephone follow-up	Telephone follow-up	Telephone follow-up	Telephone follow-up and end of study
PGI-I		✓	✓	✓		✓
ICIQ-FLUTS	✓			✓		✓
I-QOL	✓			✓		✓
Falls Diary		✓	✓	✓	✓	✓
SF-12	✓			✓		✓
Cost questionnaire			✓	✓	✓	✓

*ICIQ-FLUTS* International Consultation on Incontinence-Female Lower Urinary Tract Symptoms, *I-QOL* Incontinence Quality of Life, *PGI-I* Patient’s Global Impression of Improvement, *SF-12* 12-item Short Form Survey

Secondary outcomes such as self-reported rates of self-management for treating urinary symptoms and/or seeking professional help will be collected using identical methods from our pilot study [26]. Further, to help determine the process by which the intervention leads to significant improvement in incontinence, the International Consultation on Incontinence-Female Lower Urinary Tract Symptoms (ICIQ-FLUTS) questionnaire will measure the frequency, severity and bother from incontinence [32]. The ICIQ-FLUTS will be administered at baseline to screen participants for inclusion to the trial, and repeated at 6 months and 12 months post intervention (Table 1). The ICIQ-FLUTS diagnostic item will be used by participants to describe the type of incontinence at baseline.

#### Stigma and quality of life

The second co-primary outcome will be the difference in quality of life and stigmatization between the intervention and control groups. The 22-item Incontinence Quality of Life questionnaire (I-QOL) is a validated quality of life instrument for urinary incontinence that includes content relevant to stigma, such as “I worry about others smelling urine on me” and “I worry about being embarrassed or humiliated because of my incontinence” [33].

Exploration of stigma perceptions will be supplemented by the data collected in the qualitative interviews among a sub-group of participants immediately after, and at 1 year post intervention. Data will be collected using semi-structured one-to-one interviews. The qualitative investigation will also be used in the development of the Continence Psychosocial Impact of Assistive Devices Scale (C-PIADS), which is being developed from the original validated Psychosocial Impact of Assistive Devices Scale (PIADS). PIADS is a 26-item self-report questionnaire that includes 3 domains: competence (reflecting perceived functional capability, independence and performance); adaptability (reflecting inclination or motivation to participate socially and take risks); and self-esteem (reflecting self-confidence, self-esteem, and emotional well-being). This team is developing a modified version of this measure, C-PIADS that will evaluate the impacts of continence care product use on psychosocial constructs including perceived stigma [34, 35]. In addition to the original 26 items, the present version of the C-PIADS includes an additional 7 items, including self-consciousness, fear of being “outed,”’ social acceptance, secrecy, isolation, revealing to others, social participation and intimate relations. The C-PIADS will be administered on two occasions, after completion of the ethnographic interview, and on the telephone 1 week after the ethnographic interview.

#### Falls

The third co-primary outcome measure will be falls experienced by women exposed to the continence intervention compared to women exposed to the sham treatment 1 year postintervention. Falls will be measured as the group rate of falls at 12 months; the proportion of women who had a fall; and the proportion of injurious falls. Falls will be defined as an unexpected event in which the person comes to rest on the ground, floor or lower level [36]. Data for falls will be obtained from a falls diary with telephone reminders [37]. Previous work has shown that falls diaries pose significant advantage over other methods of capturing falls among older-aged people [37].

#### Incremental cost-effectiveness ratio and quality-adjusted life years

We will conduct both cost-effectiveness and cost-utility analysis (CEA and CUA) to explore and quantify the cost per health (or utility) gain. The primary measure of effectiveness for CEA will be presented as the incremental cost-effectiveness ratio (ICER), defined as the additional cost per participant achieving an improvement in urinary incontinence over a 1-year period. The SF-6D measure of health-related quality of life will be used to calculate utility scores for the estimation of quality-adjusted life years (QALYs) for the CUA. Derivation of the SF-6D is from item responses on the SF-12 questionnaire, a generic health- related quality of life measure, across 6 dimensions: physical functioning, role limitations due to physical health, bodily pain, vitality, social functioning, role limitations due to emotional problems, and mental health [38]. The QALY is a measure of a participant’s life expectancy, weighted by their health-related quality of life, valued on a self-reported utility score, where a score of 1 reflects perfect health and 0 is equivalent to dead [38]. The incremental cost per QALY gained will be estimated at 1 year post intervention and will be depicted in a cost-effectiveness analysis decision tree. The SF-12 questionnaire will be administered at baseline and at 6-months and 12-months post intervention, while the cost questionnaires will be administered at 3-month, 6-month, 9-month and 1-year follow-up to the study participants (Table 1).

### Sample size

The sample size calculations were not equivalent for all the co-primary outcomes of the study and, therefore, differ based on the various outcomes.

We hypothesize that the sample size required to demonstrate the effectiveness of the continence promotion intervention will be sufficient as it is powered to detect a minimally clinical important difference of 20 % in the proportion of participants globally rating their improvement in urinary symptoms as “much better” or “very much better,” with an expected change in the control group of 6 % [39]. As the design is a cluster randomized trial, we need to adjust for both the clustering and the effect of unequal cluster sizes [40]. Based on the results from the pilot study [26], an estimated average cluster size of 4 participants and an intra-class correlation coefficient (ICC) of 0.02, we would require a minimum of 14 community organizations per arm (a minimum of 56 participants per arm) to be able to detect a difference of 20 %, with 80 % power and alpha 0.05. To account for attrition, which we estimate to be around 15 %, we decided to set the recruitment target to at least 100 participants per arm.

The sample size needed for the falls outcome anticipates an average baseline fall rate per group of 30 % [26], and hypothesizes that the intervention will have an effect on the incidence rate of falls of 0.21 (30 % relative reduction). A minimum sample size of 388 per group (at least 97 community organizations per group) is needed to detect this minimally important difference with 80 % power, 5 % level of significance, an ICC of 0.02 and an average cluster size of 4.

Sample size for the economic analysis was calculated using a mapping exercise to convert a minimally important difference on the I-QOL to the SF-12 to account for the possibility that the SF-12, a generic quality of life measure, may be insufficiently sensitive to reflect important differences in incontinence-related quality of life [39]. An improvement of 6.65 points on the I-QOL scale best matched an improvement (as much better or very much better) on the PGI-I, which we defined as a minimally important difference. Then, using a Monte Carlo simulation, we calculated that using this change cut-off, a detectable difference of 4.74 on the I-QOL between the intervention and control groups, would be statistically significant (at 0.05 alpha level) in at least 80 % of simulations when each group comprises at least 500 participants, representing a gain of 0.0126 in SF-6D utility scores.

Taking into account the sample size calculation for all co-primary outcome measures, we therefore aim to recruit the participants according to the largest sample size that will be sufficient for sub-group analyses (*n* = 500 participants per group) – a total of 1000 participants.

## Data analysis

### Co-primary outcomes

Differences in baseline characteristics between the groups will be determined using descriptive statistics (means, proportions), to assess any imbalance in potential confounders. All analyses will be done on an intention-to-treat basis. To assess improvements in incontinence, we will estimate the unadjusted risk difference (prevalence of the outcome) and 95 % confidence interval (CI) via generalized estimating equations (GEEs) with an identity link and an exchangeable correlation structure to account for possible correlation between women in the same organization [41]. The effects of various potential confounders in the groups at baseline will be explored because of the possibility for selection bias in an open trial. To adjust for imbalance in potential confounders, additional analyses will be conducted using multivariate logistic regression estimated via GEE with an exchangeable correlation structure. As a sensitivity analysis, we will repeat the analysis on a per protocol basis that will include participants who adhered to the protocol and follow-ups.

Incontinence-specific quality of life will be assessed using the Incontinence Quality of Life Instrument (I-QOL). The 22 items in the I-QOL will be summed and then transformed to a 0 to 100 scale for greater interpretability, with the higher scores representing greater quality of life. The I-QOL subscales (avoidance or limiting behaviors, psychological impacts, social life impact, and social embarrassment) will be scored in an identical manner. In an ad hoc analysis, crude effect sizes, examining change in scale scores at each assessment within participant group (intervention and control), will be calculated and evaluated using Cohen’s *d* criteria (i.e., 0.2–0.49 is small, 0.5–0.79 is moderate, and 0.8 and above is large). Multivariate linear models will be used to compare each I-QOL subscales score reported by the intervention group to the control group, with the control group as the referent and participants clustered by organization. Age at baseline and other potential confounders will be included as covariates in all adjusted models.

Fall rates in the intervention and the control groups will be analyzed as follow: (1) the incidence rate of falls, using Poisson regression to allow for clustering of falls by the same participant, (2) the proportion of patients who had one or more falls versus no falls, using logistic regression analyses, and (3) the proportion of injurious falls, using logistic regression analyses. Each analysis will use patient-level data that will be clustered by organization and analyzed via GEE with an exchangeable correlation structure and a log and a logit link for Poisson and logistic regression analyses respectively. Negative binomial regression will be used to model the incidence rate of falls, if there is evidence of over-dispersion of the data using Poisson regression. Multivariate analyses will adjust for any confounding variables that may affect the relationship between incontinence and falls.

### Analysis of the qualitative sub-study on stigma

The interview data on stigma will be analyzed using the principles of content description. The goal of content analysis is to generate a group of concepts that describes primary research questions. All text that addresses the fundamental research questions will be coded, whereby relevant text will be assigned a “heading” in the margin of the transcript. Headings will be reviewed, and categories of headings created. In the final step of content analysis, investigators will identify meaningful patterns within and across interview transcripts, and prepare general descriptors of the data. For this step, we will upload the data into ATLAS-ti, a software program designed to aid in the analysis of large bodies of text.

### Economic analysis

**Fig. 3:**
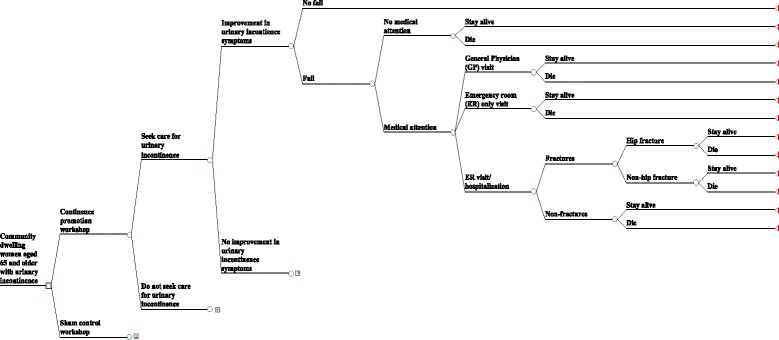
Decision tree model for the cost-effectiveness analysis. Legend: decision-tree model for the cost-effectiveness analysis. The square indicates group randomization; the circles indicate chance nodes; the triangles indicate terminal nodes

In this analysis we will assess the cost of the continence promotion program for senior women with incontinence compared to the sham control group. The study will involve constructing a decision analytic model through which we will estimate the cost-effectiveness and cost-utility of the intervention. This will take the form of a decision tree and will be based directly on the prevalence for the global rating of improvement (as “much better” or “very much better”) in urinary incontinence as the outcome (Fig. 3). Each of the branches in the decision tree represents a set of actions or strategies under assessment and will be populated with data on probability, costs and utilities obtained from data collection in the study. The decision tree remains identical for every option in the model, although probabilities change.

Data and parameter estimates specific to Canada will be used to calculate the ICERs, first from a societal perspective and then from a healthcare system perspective. ICERs for the healthcare system perspective as base case will be of interest to site-specific health policy makers for resource allocation decisions, should the continence promotion intervention be found effective. Critically, the ICER from a societal perspective will facilitate discourse on the full opportunity cost of individual pad use in the context of the selected site. The results of the CEA will be expressed as cost per percentage improvement in the prevalence of urinary incontinence as measured by PGI-I and the results of the CUA will be expressed as cost per QALY gained. The ICER from the CEA will be comparable with other continence intervention programs using the same outcome measure (improvement in urinary incontinence), while QALYs from the CUA is a more standardized measure (not specific to the outcome measure) and will be comparable more broadly.

A probabilistic sensitivity analysis will be undertaken using the uncertainty estimates, in which the key model’s parameters will be replaced with plausible values drawn from specified probability distribution assigned to those parameters. Uncertainty surrounding the cost-effectiveness and cost-utility results will be provided by plotting replications on the cost-effectiveness and cost-utility plane and reviewed in cost-effectiveness and cost-utility acceptability curves.

## Discussion

This study aims to evaluate the impact of a continence promotion intervention on community-dwelling older women with untreated incontinence. Previous research suggests that an intervention combining constructivist learning and an evidence-based self-management tool is effective in reducing urinary symptoms. [26] The current study will investigate whether the benefits of this continence promotion intervention extend to a reduction in falls and stigma, improvements in quality of life and social participation, and gains in healthy active life expectancy. Generalizability to different cultural settings will be achieved by testing the intervention across three countries in both English and French to determine its effect on multiple co-primary outcomes.

### Strengths

The strengths of this study include the robust randomized controlled trial design with baseline and long-term follow-up, the quantitative and qualitative mixed analysis and the use of multiple measures as primary outcomes. Additionally, the international platform for this study will facilitate rapid accrual of sample size and external validity at minimum cost, while meeting the needs of regulatory agencies in different jurisdictions. The synergies enabled by the intervention have the potential to produce a paradigm shift in attitudes toward incontinence in older women, and among health professionals empowered to address multimorbidity in an aging population. Although incontinence has been associated with falls, to date no trial exists that causally links reductions in falls to exposure to a continence promotion intervention that improves urinary symptoms. Furthermore, this is the first trial to assess the impact of incontinence symptom improvement, fall reduction and quality of life on QALYs and healthy active life expectancy in older women.

### Limitations

There are a few limitations to our study. Many of our outcome measures are by self-report, though supplemented with objectively measured data whenever possible. We attempted to use a bladder diary to measure improvements in incontinence during our pilot study [26] in the UK, however, a large proportion of unreliable or missing data was collected. Further, for incontinence severity of less than once every 2 days, the responsiveness of this measure is poor [42]. We acknowledge the possibility of recall bias for the baseline measures and importantly, the diary falls entry. However, we tried to limit this bias by implementing telephone follow-up reminders.

Post-randomization recruitment bias is also a possibility for participants who have not been screened for incontinence prior to randomization of the clusters [27].

### Knowledge translation

Knowledge translation is assured as our recruitment strategies reach out directly to community organizations and their constituents. The results of this study will be communicated to community stakeholders and the general public as an incontinence awareness initiative. Our ultimate goal will be to contribute to discussions around public health social policy change to support a more proactive society-wide management of incontinence. The progress of the study, interim and final results will be presented at relevant national or international conferences. Manuscripts will be submitted for publication in peer-reviewed journals. The aim is to make the intervention accessible in a train-the-trainers format to community organizations and primary care institutions after completion of the study.

### Potential impact

Incontinence increases with age and is strongly associated with reduced self-esteem, social isolation, depression, and a higher likelihood of dependency and institutionalization. The current lack of social policies and public health initiatives surrounding community-based continence promotion strategies offers a fertile ground on which to build this study and a stepping stone for developing continence expertise in other related settings such as hospitals and long-term care. Despite the high prevalence of incontinence in seniors, there is a surprising lack of evidence-based public health campaigns to improve continence among older women and to reduce the stigma, negative beliefs and loss of functional independence associated with incontinence.

This study will use a novel constructivist group intervention combined with innovative evidence-based self-management tools for older community-dwelling women with incontinence, with the aim of increasing disability-free lifespan and quality of life. The economic analysis will determine the costs incurred by the continence promotion intervention and facilitate a realistic budget impact assessment that can be used to supplement sustainable interventions for healthy aging.

Evidence linking continence promotion to fall reduction and improvements in healthy active life expectancy will inform future public policy and fall prevention programs.

## Trial status

The trial is currently recruiting participants and is approximately 60 % complete at the time of submission.

## Abbreviations

CACTUS-D: Continence Across Continents To Upend Stigma and Dependency
CEA: cost-effectiveness
CI: confidence interval
C-PIADS: Continence Psychosocial Impact of Assistive Devices Scale
CUA: cost-utility analysis
GEE: generalized estimating equations
ICC: intra-class correlation coefficient
ICER: incremental cost-effectiveness ratio
ICIQ-FLUTS: International Consultation on Incontinence-Female Lower Urinary Tract Symptoms
I-QOL: Incontinence Quality of Life questionnaire
PIADS: Psychosocial Impact of Assistive Devices Scale
PGI-I: Patient Global Impression of Improvement
QALYs: quality-adjusted life years
RCT: randomized controlled trial
SF-6D: 6-dimensional derivative of the 12-item SF-12
SF-12: 12-item Short Form Health Survey
UK: United Kingdom
